# Vibrational Spectroscopy in Assessment of Early Osteoarthritis—A Narrative Review

**DOI:** 10.3390/ijms22105235

**Published:** 2021-05-15

**Authors:** Chen Yu, Bing Zhao, Yan Li, Hengchang Zang, Lian Li

**Affiliations:** 1 School of Pharmaceutical Sciences, Cheeloo College of Medicine, Shandong University, Wenhuaxi Road 44, Jinan 250012, China; y17865190891@163.com (C.Y.); zhaobing911@163.com (B.Z.); zanghcw@126.com (H.Z.); 2 NMPA Key Laboratory for Technology Research and Evaluation of Drug Products, Shandong University, Wenhuaxi Road 44, Jinan 250012, China; 3 Institute of Materia Medica, Shandong First Medical University and Shandong Academy of Medical Scinces, Jinan 250062, China; liyan091022@126.com; 4 Key Laboratory of Chemical Biology (Ministry of Education), Shandong University, Wenhuaxi Road 44, Jinan 250012, China; 5 National Glycoengineering Research Center, Shandong University, Wenhuaxi Road 44, Jinan 250012, China

**Keywords:** osteoarthritis, vibrational spectroscopy, near-infrared spectroscopy, infrared spectroscopy, Raman spectroscopy, early diagnosis

## Abstract

Osteoarthritis (OA) is a degenerative disease, and there is currently no effective medicine to cure it. Early prevention and treatment can effectively reduce the pain of OA patients and save costs. Therefore, it is necessary to diagnose OA at an early stage. There are various diagnostic methods for OA, but the methods applied to early diagnosis are limited. Ordinary optical diagnosis is confined to the surface, while laboratory tests, such as rheumatoid factor inspection and physical arthritis checks, are too trivial or time-consuming. Evidently, there is an urgent need to develop a rapid nondestructive detection method for the early diagnosis of OA. Vibrational spectroscopy is a rapid and nondestructive technique that has attracted much attention. In this review, near-infrared (NIR), infrared, (IR) and Raman spectroscopy were introduced to show their potential in early OA diagnosis. The basic principles were discussed first, and then the research progress to date was discussed, as well as its limitations and the direction of development. Finally, all methods were compared, and vibrational spectroscopy was demonstrated that it could be used as a promising tool for early OA diagnosis. This review provides theoretical support for the application and development of vibrational spectroscopy technology in OA diagnosis, providing a new strategy for the nondestructive and rapid diagnosis of arthritis and promoting the development and clinical application of a component-based molecular spectrum detection technology.

## 1. Introduction

Osteoarthritis (OA) is one of the major diseases affecting public health, and it is caused by both the mechanical and biological degradations of cartilage [1,2]. The 2017 Global Burden of Disease study shows that the annual number of OA patients is increasing, yet there remains no effective medicine to treat it [3]. OA is characterized by joint swelling, pain, and stiffness, causing physical pain to and placing a heavy financial burden on patients [4]. MacDonald et al. reported that the average age of people diagnosed with OA is 47.6, which is 7.7 years later than the onset of symptoms [5]. A Canadian study reported that patients with an OA score greater than or equal to 55 (severe disability) cost 3.5 times more than patients with an OA score of 15 (low severity) [6]. Most patients usually choose to see a doctor after they have obvious symptoms, and the general treatment of OA is often to address the corresponding symptoms, rather than to fundamentally cure the disease [7].

Many physiological and pathological changes may appear in the early stage of OA, which are the starting point for early diagnosis. OA is mainly caused by the inability of chondrocytes to maintain homeostasis between the synthesis and decomposition of intracellular and extracellular components, resulting in the inflammation of the synovium and joint capsule [8]. Moreover, various pathological changes may combine and worsen, leading to cartilage degeneration, subchondral osteosclerosis, angiogenesis, innervation, and other pathological phenomena. The extracellular matrix of chondrocytes is mainly composed of collagen, noncollagen proteins (glycosaminoglycan (GAG), proteoglycan (PG), cartilage oligomer matrix protein (COMP)), hyaluronic acid (HA), fibrin protein, follistatin-like protein 1 (FSTIL-1), glycoproteins, lipids, and water [9]. A number of these proteins are often used as biomarkers for the diagnosis of OA. GAG is the main component of the cartilage matrix. GAG in cartilage is cleaved by proteolytic enzymes, such as matrix metalloproteinases (MMPs) and aggregase, which decrease the expression level of GAG and lead to cartilage destruction. Therefore, GAG is often used as a marker of cartilage destruction in OA [10]. As an important component of synovial fluid (SF), HA can increase joint smoothness, thereby reducing cartilage wear, and it has been shown to be associated with the radiological progression of OA [3]. Subchondral bone can provide mechanical and nutritional support for cartilage. Small molecule diffusion experiments revealed that there is a direct molecular signaling pathway between cartilage and subchondral bone [11], indicating that changes in the microenvironment of subchondral bone may directly or indirectly affect cartilage metabolism. The quality and hardness of bone in patients with OA are decreased, making it more likely for some injuries to occur [12] and eventually leading to the production of osteophytes. In subchondral bone, markers such as the mineral-to-matrix ratio, mineral maturity/crystallinity, relative carbonate content, or relative tissue water content/porosity can provide signs of early OA [13]. The detection of changes in these markers is beneficial for an early diagnosis, which not only improves the treatment effect of OA for patients, but also greatly reduces treatment costs [14]. Considering the importance of an early diagnosis of OA, in this review, we aimed to summarize and analyze the vibrational spectroscopy used in early OA diagnosis to provide theoretical support for its application and development.

The diagnosis of OA primarily involves imaging and laboratory tests. The conventional imaging tests used include magnetic resonance imaging (MRI) [15,16,17,18], optical coherence tomography (OCT) [18,19,20,21], ultrasonic diagnosis [22,23,24,25,26], and X-ray [27,28,29]. Their specific applications are shown in Table 1. X-ray is the most accessible tool for assessing OA: it can show damage and other changes related to OA to confirm its severity according to different grading schemes, such as the Kellgren–Lawrence grading scheme [30]. MRI, which does not use radiation, is more expensive than X-ray but can provide better images of cartilage and other structures to detect early abnormalities in OA [16]. OCT is used to generate cross-sectional images of articular cartilage [19], and it can provide quantitative information on the state of articular cartilage, especially for OA caused by changes in collagen structure [31]. In terms of laboratory tests, the main method is joint aspiration, which comprises a needle being inserted into the joint, and fluid is extracted for further analysis to determine the stage of OA, which can help to rule out other forms of arthritis [32].

**Figure 1:**
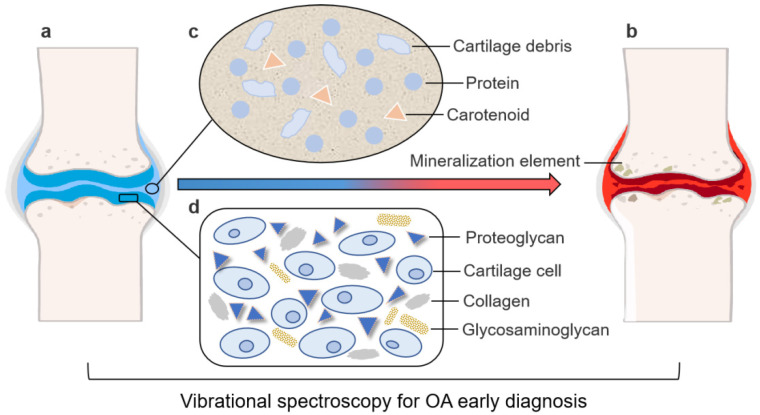
Vibrational spectroscopy is used for early OA diagnosis by detecting changes in the composition and morphology of articular cartilage and changes in synovial fluid (SF) and subchondral bone. The figure depicts (**a**) a healthy joint and (**b**) an OA-affected joint. In an OA-affected joint, the SF composition changes, articular cartilage becomes thinner and its composition changes, and the mineralization element in the subchondral bone increases. The main biomarkers of OA in (**c**) SF and (**d**) articular cartilage are also shown. Vibrational spectroscopy can detect the changes of these components for early OA diagnosis.

The methods mentioned above are commonly used in clinical practice but are often time-consuming, expensive, and even destructive. The development of rapid and nondestructive methods is urgent [19,29]. As a promising technique, vibrational spectroscopy has received increasing attention from researchers worldwide. A vibrational spectrum is generated by the vibration of molecules or atomic groups caused by electromagnetic radiation, and vibrational spectroscopy includes a variety of techniques, such as near-infrared (NIR), infrared (IR), and Raman spectroscopy [33]. As shown in Figure 1, NIR/IR/Raman spectra of SF, articular cartilage, and/or subchondral bone were collected to compare the spectral differences between healthy and OA-affected joints. The spectra of SF indicate that the content of protein increased and the content of carotenoid decreased in OA-affected joints [34]. Furthermore, the spectra of articular cartilage characterized changes in the contents of GAG and PG, as well as the orientation of collagen fibers in joints [35]. By analyzing the spectra of subchondral bone, it was concluded that mineralized components increased in OA-affected joints [36]. Extracting and analyzing effective spectral information can help to distinguish OA-affected joints from healthy ones, thereby improving the likelihood of early OA diagnosis. In this review, these new vibrational spectroscopy methods for OA diagnosis are introduced to provide theoretical support for their application and development in relation to the early diagnosis of OA.

## 2. NIR Spectroscopy

**Figure 2:**
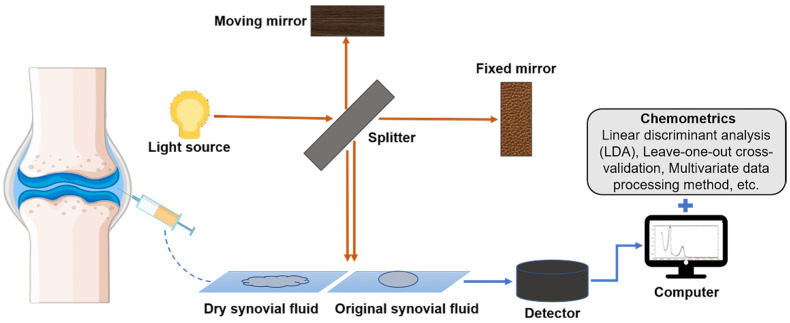
SF is analyzed by NIR spectroscopy. The SF extracted from the joint capsule is dried and either membrane-formed or examined directly. The light source is divided into two beams by the splitter, which are then reflected onto two mirrors—one is a fixed mirror and the other is a moving mirror. After the light is reflected, it is recombined and sent to the sample, and then the change of the light beam after the reflection and absorption of the sample is detected. Finally, the spectral data are analyzed with the help of chemometrics.

NIR spectroscopy reflects the overtones and/or combination bands of stretching and bending vibrations of C–H, N–H, and O–H bonds ranging from 12,500 to 4000 cm^−1^ [37]. As shown in Figure 2, when a beam of NIR light with continuous wavelength irradiates to the sample, if the vibrational/rotational frequency of some groups in the sample is consistent with that of the NIR light, the molecule will absorb energy for energy-level transition, and the light at that wavelength is absorbed [38]. The sample can then be analyzed according to the transmitted or reflected light. NIR spectroscopy has been widely used in the agriculture [39,40,41,42], food [43,44,45,46], chemistry [47,48,49], and pharmaceutical fields [50,51,52,53] because it is fast, accurate, nondestructive and labor-saving. However, a limitation of this method is that the spectra are composed of wide overlapping bands [54,55], such that it is difficult to find specific peaks attributed to the complicated component. Chemometrics is a powerful tool for analyzing the NIR spectra and can improve the spectral resolution to help extract useful information from the spectra [56]. With the help of chemometrics, NIR spectroscopy has appeared in OA diagnostic research owing to its fast and nondestructive characteristics [57]. Although most of the work of NIR spectroscopy in OA diagnosis, including SF and tissue analysis, is still restricted to laboratory-scale research [58], it is becoming a useful tool for the diagnosis of OA and has good development prospects.

### 2.1. Application of NIR Spectroscopy on OA SF Analysis 

Some studies have shown changes in the composition and concentration of SF metabolites in OA [59,60]. Therefore, SF is useful for the investigation of OA based on chemical composition and physical properties. There are two main methods for SF spectra collection: The first is to dry the SF onto a film and then to obtain the reflection spectrum for analysis. This can identify arthritis with a classification rate greater than 95% [61]. The second method is to obtain the SF spectrum through the transmitting module. Shaw et al. [58] collected the NIR spectra of SF and then divided the spectra into three categories by linear discriminant analysis (LDA), which is a kind of subjective measurement and classification based on a series of biochemical or biological diseases, leading to SF physical change. Their results showed that the best classification results were obtained when the spectral range was limited to 2000–2400 nm. This research demonstrated that NIR spectroscopy can determine the type of arthritis and the feasibility of severity ratings by SF analysis, showing that it is a powerful tool for the diagnosis of OA based on SF analysis. However, the application of this technology in SF analysis requires further research, which may help provide new hypotheses to explain the mechanism of OA in the future.

### 2.2. Application of NIR Spectroscopy on OA Cartilage Analysis

Normal hyaline cartilage contains 70%–80% water, mainly in the form of binding to GAG. In the early stage of OA, cartilage deformities often manifest as complex changes in the matrix, including GAG, moisture, and collagen [62]. Hofmann et al. [63] compared the degree of recognition of OCT, arthroscopy, MRI, and NIR spectroscopy for early OA cartilage change and found that only the result of NIR spectroscopy showed good correlation with the Knee Injury and Osteoarthritis Outcome Score (KOOS). Afara et al. [57] analyzed the NIR spectra of articular cartilage in mice with OA in four stages (1, 2, 4, and 6 weeks), and the spectral data showed that the spectral intensity increased over time, mainly due to changes in the cartilage moisture content; this showed that the method could detect early manifestations of cartilage degeneration. In addition, they applied principal component analysis (PCA) and multiple scattering correction (MSC) to process the two main spectral bands at 8547–10,361 cm^−1^ and 6411–6496 cm^−1^ and predict the Mankin score [64,65]. They also designed a genetic algorithm-based model to evaluate GAG content, which further demonstrated the power of NIR spectroscopy in OA diagnosis by detecting tissue integrity and GAG content.

The main feature of OA is cartilage degeneration, and PG and GAG are the key components of cartilage [66], so their effective detection is crucial for early diagnosis of OA. Palukuru et al. [67] identified 20–60 μm thickness of cartilage tissue as most suitable for analysis based on collagen and PG at 1336 and 856 cm^−1^ for linear absorption band intensity changes. They used partial least squares (PLS) modeling in the spectral range of 4000–6000 cm^−1^ to predict the relative content of collagen and PG, as well as the proportion of collagen, from NIR spectroscopy. The error of this method was reduced to 6%, which ultimately improved the accuracy of NIR spectroscopy for cartilage composition quantification.

In addition, a direct manifestation of OA is the reduction and thinning of cartilage [68]. Sarin et al. [69] combined arthroscopy with NIR spectroscopy to evaluate the cartilage thickness in vivo and determine PG content and the collagen orientation angle. Using a one-dimensional neural network, they demonstrated the clinical potential of NIR spectroscopy for evaluating cartilage thickness and composition structure. Afara et al. [70] combined NIR spectroscopy with support vector machines (SVMs), deep neural networks (DNNs), logistic regression (LR), and other methods to distinguish the integrity of cartilage. Their study indicated that the SVM model can distinguish anterior cruciate ligament transection (ACLT) from non-operationally operated control (CNTRL) samples, the DNN model can discriminate between different types of OA, and LR can differentiate between contralateral (CL) and CNTRL samples. NIR spectroscopy, in combination with machine learning techniques, can provide a powerful tool for the classification of cartilage integrity [71], with the potential to accurately distinguish between normal and early osteoarthritic cartilage [72,73].

## 3. Infrared Spectroscopy

The principle of IR is similar to that of NIR spectroscopy, but the difference lies in the acquisition bands used by the methods. IR spectroscopy technology is used to investigate interactions between molecules in the range of 4000–400 cm^−1^ [74]. It can be used to analyze the characteristic information of absorbed substances by reflecting IR light. Each molecule has a unique IR absorption spectrum, which is determined by its composition and structure, and this can be used for structural analysis and identification [75]. Fourier-transform IR (FTIR) spectroscopy is a simple method used to investigate the composition of macromolecules in biological samples, and it can be analyzed in situ by a probe, so it has advantages in the study of organisms and for early diagnosis of OA [76]. FTIR microspectroscopy (FTIR–MS) comprises an FTIR spectrometer and a traditional optical microscope to achieve chemical imaging, with which the spatial distribution and structure of various biochemical components can be identified [77]. Due to the complexity of the data structure, the required information cannot be revealed without powerful data analysis methods. Therefore, the application of IR spectroscopy in the biomedical field usually requires the combination of advanced multivariate data analysis methods [78]. At present, IR spectroscopy, especially FTIR spectroscopy, has shown great application prospects in the early diagnosis of OA, mainly including SF analysis and tissue analysis.

### 3.1. Application of IR Spectroscopy to SF Analysis in OA Subsection

The analysis of changes in SF by IR spectroscopy is also an effective method for the early diagnosis of OA. Eysel et al. [79] used LDA and leave-one-out cross-validation to classify 239 SF membrane spectra from 86 patients. Through multivariate analysis, the spectra were successfully divided into four categories, which were consistent with the clinical diagnosis (96.5% correct classification). By collecting the IR spectrum of joint fluid, Hou et al. [80] established an effective diagnostic model, indicating that IR spectrum technology combined with a multivariate data processing method can be used as a simple and effective method for OA diagnosis. In addition, IR spectrum data based on serum and articular fluid were shown to differentiate between samples of healthy dogs and dogs with OA, indicating that IR spectroscopy combined with multivariate data analysis is a simple and accurate diagnostic method for OA.

### 3.2. Application of FTIR Imaging on Articular Cartilage Analysis in OA 

Some manifestations of OA, including dissolution, thinning, compositional changes, mineralization, and subchondral bone thickening [81], can be detected with traditional diagnostic techniques. By using FTIR imaging (FTIRI), both the corresponding spectral image of each pixel and the complete pathological joint image can be obtained, so the diagnostic analysis can be carried out from both the macro and micro perspective. Yin et al. [82] used FTIRI to examine the content of collagen and PG, along with the change of depth in healthy cartilage and OA cartilage. Their results showed that the deep dependence of PG levels was different between healthy and OA-affected subjects. Therefore, the FTIRI technique could be used to detect changes in cartilage composition related to OA for early diagnosis. However, the data analysis of the IR spectrum is complex, and accurate data processing methods are needed to optimize data analysis and gather information. Rieppo et al. [83] carried out optimal variable selection for FTIR spectral analysis of articular cartilage composition, and successfully determined the major components of AC, PG, and collagen using multiple regression.

The IR spectra absorption peaks of PG and collagen often overlap, and a second derivative is normally applied to improve the resolution of the overlapping peaks. Rieppo et al. [84] provided a practical approach for the analysis of articular cartilage composition by a second derivative analysis of collagen protein and polysaccharide. David-Vaudey et al. [85] collected FTIRI spectral images on the surface, middle, and deep layers of samples and then used a reference spectrum to perform Euclidean distance mapping and quantitative PLS on type II collagen and chondroitin sulfate (CS). Their results showed that the FTIRI results were correlated with the Mankin scoring system based on histology, and PLS analysis showed that the relative concentrations of collagen and PG in OA cartilage were relatively low.

Fisher discriminant analysis (FDA) establishes a discriminant function through covariance analysis to classify data sets rapidly. The discriminant principle of FDA is to ensure maximum covariance between different categories and minimum covariance within a category. Mao et al. [86] extracted FTIR images and performed a PCA of IR spectra, and then established a model to identify articular cartilage samples by FDA. Their results showed that all healthy cases in the prediction group were correctly identified, and only a few individual OA cases were misdiagnosed. Similarly, Zhang et al. [87] used FTIRI and PLS–DA methods to identify healthy cartilage and OA cartilage. In the calibration and prediction matrix, the recognition rates of healthy cartilage and injured cartilage were 100% and 90.24%, respectively. Mao et al. [88] predicted that both FTIRI–PLS–DA and FTIRI–PCA–FDA integration technologies would become tools for the microscopic identification of healthy cartilage and OA cartilage specimens and the diagnosis of cartilage lesions. Oinas et al. [78] used cluster analysis to investigate human articular cartilage samples with different histological levels of OA. Through cluster analysis, the IR spectrum can be used to compare multiple images in order to quantitatively and qualitatively detect changes in the surface and deep layers of articular cartilage at the early stage of OA.

### 3.3. Application of FTIR in OA In Situ Analysis

One advantage of FTIR is that the IR fiber-optic probe (IFOP) is coupled to the FTIR spectrometer, which provides in situ full-tissue spectral acquisition and has the potential to evaluate OA in vivo. Johansson et al. [89] measured the thickness of articular cartilage based on the principle of broadband diffuse light reflection and conducted an in vitro study on the thickness of articular cartilage in multiple parts of human knee condyles with the specific goal of arthroscopic integration. An exponential model was used to compare the estimated value with the reference cartilage thickness value (obtained after section). A two-dimensional Monte Carlo simulation analysis was used to estimate the thickness of human knee cartilage, and the thickness distribution of cartilage on the joint surface could be visualized. This method provides support for research in the field of arthroscopy. Hanifi et al. [90] applied IFOP to collect spectra from normal and degraded areas of OA and established a multiplex PLS method based on the second derivative to predict the Mankin score of histology with an accuracy of 72%. Similarly, West et al. [91] found that type II collagen degradation was associated with chondrogenic degeneration, which could be used to monitor small changes associated with early cartilage degeneration. This demonstrated that IFOP could be used to perform in situ determination of cartilage integrity during arthroscopy. Yang et al. [92] used FTIR–MS spectroscopy to detect changes in the tibial articular subchondral bone in guinea pigs with increasing age, and their results showed that the ratio of amide III to amide II was consistent at different ages. At the molecular level, this research provides reliable pathological information for subchondral bone histology of OA and technical support for the early diagnosis of OA. However, the novel attenuated total reflectance–mid-IR–hollow optical fiber (ATR–MIR–HOF) probe has more advantages than traditional FTIR and ATR–FTIRI techniques. ATR–HOF–FTIR can obtain reliable ATR–IR spectra without the need for sample pretreatment. Its detection of articular cartilage damage shows that the ATR–MIR–HOF probe can easily detect changes in a small target region in situ and obtain spectral information related to changes in the major components of articular cartilage with good repeatability [93].

## 4. Raman Spectroscopy

Raman spectroscopy is a vibrational spectroscopy technology based on the Raman scattering principle. Rayleigh scattering and Raman scattering of different wavelengths are generated when a laser shines on a sample [94]. Rayleigh scattering is consistent with the wavelength of the incident light, while Raman scattering is different from the frequency of the incident light and can reflect specific intermolecular vibrations, such as C−C, C=C, C−O, and C−H [95]. Therefore, the characteristic information of the material can be analyzed by collecting Raman spectrum lines. Over recent years, Raman spectroscopy technology has continued to be developed, and researchers have combined Raman spectroscopy technology with a variety of other technologies to make detection technologies more effective, such as confocal Raman microscopy [96], Raman imaging technology [97,98], resonance Raman technology [99], surface-enhanced Raman spectroscopy (SERS) technology [100], and so forth. Raman spectroscopy technology is widely used in the fields of medicine [101], pharmaceuticals [102,103,104], cosmetics [105,106], carbon materials [107,108], geology [109,110], and life sciences because of its ability to rapidly analyze chemical structures in a nondestructive manner and its powerful imaging functions. Compared with other spectroscopic techniques, Raman spectroscopy has improved analytical performance for samples in aqueous solutions, biological tissues, and cells because the Raman signal of water is very weak. Increased research attention is being paid to the use of Raman spectroscopy for the diagnosis of OA through the analysis of changes in SF, subchondral bone, and articular cartilage, among others.

### 4.1. Application of Raman Spectroscopy on OA SF Analysis

The early occurrence of OA is accompanied by the degeneration, dissolution, and fragmentation of articular cartilage. Raman spectroscopy can be used to detect changes of SF in joints for the early diagnosis of OA. Esmonde-White et al. [111] conducted a study on the correlation between NIR–Raman spectroscopy of SF and radiological scores of knee joint injury in patients with OA. Changes in the SF composition of patients were studied by NIR–Raman spectroscopy, and data were analyzed by *K*-cluster analysis. The results showed that the spectral signals of SF in patients with different grades of OA differed. HA, a main component of SF, is an important biomarker in the diagnosis of OA [112,113]. With the development of OA, the amounts of amide III (1250 cm^−1^) and C–C, C–O bonds (1155 cm^−1^) in HA increased [114], which showed the potential of Raman spectroscopy in relation to the diagnosis of OA by SF analysis.

Researchers have been searching for biomarkers in SF that can be used to diagnose OA earlier and with greater reliability. However, the composition of SF is complex, and it is difficult to effectively study its single components, so it requires the development of signal enhancement techniques [115]. As an important component of SF, HA has attracted the attention of researchers. Mandair et al. [116] found that SERS could greatly enhance the signal intensity of HA, such that the minimum detectable concentration could be as low as 0.5 mg/mL. At the same time, effective protein removal techniques, including the trichloroacetic acid precipitation method, centrifugal method, and droplet deposition method, can effectively remove the influence of protein on Raman spectrum to observe HA more clearly. Bocsa et al. [34] studied SF using resonance Raman technology combined with SERS. They found low carotenoid levels in advanced OA patients, and using a PCA–LDA analysis method to classify OA patients, they achieved an accuracy of 100%. With the development of various Raman enhancement techniques, Raman spectroscopy analysis of biomarkers in SF has become a good prospect for the early diagnosis of OA.

### 4.2. Application of Raman Spectroscopy in Subchondral Bone Analysis in OA

As one of the main manifestations of OA is the proliferation of subchondral bone, OA can be diagnosed by analyzing changes in it [117,118]. Researchers compared the internal and external Raman spectroscopies of knee subchondral bone in OA patients and healthy people. Using multivariate analysis, they found that both the internal and external components of subchondral bone in OA patients were altered, and there were significant spectral differences between OA and healthy people (*p* < 0.001). Differences primarily manifested in the phosphate band (954 and 966 cm^−1^), amide I (1668 and 1685 cm^−1^), and shoulder (941 cm^−1^), and the proportion of type I collagen chain in OA was significantly higher [119,120,121].

The development of OA can be distinguished by observing changes in subchondral bone composition [122]. Das Gupta [123] investigated subchondral bone and changes of calcified cartilage-specific biochemical composition in OA with NIR–Raman spectroscopy technology. *K*-means clustering analysis and hierarchical cluster analysis (HCA) showed that calcified knees (CCs) were more mineralized than the subchondral bone plate (SBP), and that the mineral had a higher crystallinity. The degree of mineralization of the two tissues began to change from the early stage of OA. In the late stage of OA, the mineral crystals were rich in carbonate, but the overall mineralization had decreased. The Raman spectra of subchondral bone collected during in situ analysis are often doped with cartilage or even cancellous bone signals. Esmonde-White et al. [124] used Raman arthroscopy to conduct an in situ analysis of OA and compared the Raman spectra of the articular surface with the standard spectra of isolated articular cartilage and subchondral bone in order to study the influence of cartilage thickness on the Raman spectra of articular cartilage. The in situ spectrum reflected the mixed signal of articular cartilage and subchondral bone that as the cartilage becomes thicker, the spectral expression of subchondral bone and cancellous bone decreases. This study provides theoretical support for the further study of the Raman probe in relation to diagnosis of OA.

### 4.3. Raman Spectroscopy Analysis of Articular Cartilage in OA

**Figure 3:**
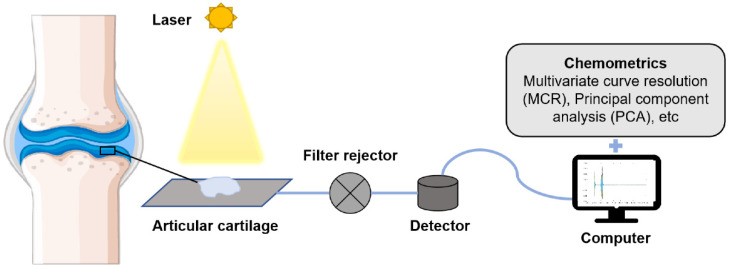
Articular cartilage is detected by Raman spectroscopy. The laser light source irradiates the articular cartilage, and the scattering light is generated through the scattering of the sample. Raman scattering light is obtained by the filter, and the Raman spectrum is presented after its detection and processing by a detector. Spectral data are analyzed with the help of chemometrics.

OA is mainly manifested by the degeneration of articular cartilage and reduced elasticity, thinning, or even dissolution and fragmentation of articular cartilage, leading to the decrease of joint lubricity [122]. As shown in Figure 3, OA can be diagnosed and analyzed by observing changes in the articular cartilage with Raman spectroscopy. The main components of articular cartilage are GAG and collagen [123], and changes in them can be used as indicators for the early diagnosis of OA. Mason et al. [125] used Raman multivariate curve resolution (MCR) to analyze the cartilage surface, and their results showed that Raman MCR could accurately quantify the cartilage subcomponent distribution on the entire surface, with a depth of up to 0.5 mm. Jensen et al. [126] collected Raman spectra from the cartilage tissue model and found that there were slight differences in the spectra of different tissue regions, which represented the orientation of collagen fibers, proving that polarized Raman spectroscopy can distinguish between collagen fiber orientations in the cartilage explant model system. In another study, Lim et al. [127] examined real pig cartilage, and their spectral results showed that the amide III spectral band had a red shift from 1264 to 1274 cm^−1^, reflecting the compression possibility of CN vibration in collagen fiber. Furthermore, a decrease of 1042 cm^−1^ in the GAG-related pyran saccharide band indicated a decrease in GAG content in OA patients. Kumar et al. [128] classified cartilage according to the definition provided by the International Cartilage Maintenance Society (ICRS) of OA, and then collected Raman spectra and analyzed them using PCA. Their results showed that, in the early stage of OA, the content of helical collagen is significantly increased, while only in the late stage of OA could an obvious difference in GAG be shown. De Souza et al. [129] used Raman spectroscopy to detect molecular changes related to OA induced chemically and by treadmill exercise. Their results showed that both OA experimental models significantly increased the Raman ratio of tissue remodeling associated with mineralization. Compared with the chemical induction model, the content of phenylalanine in the treadmill exercise induction model was significantly lower and the crystallinity was higher. Their study showed that Raman spectroscopy can not only diagnose and detect cartilage damage at the molecular level, but also monitor and analyze subchondral bone and cancellous bone in the pathogenesis of OA.

Raman spectroscopy can be analyzed at the cellular level for early diagnosis of OA. Kumar et al. [130] showed that Raman spectroscopy can distinguish between stages of OA. According to their analysis, the contents of amide I (1612–1696 cm^−1^) and protein decrease with increasing severity of OA, and the intensity of the spectral band at 1302–1307 cm^−1^ with the peak value at 1304 cm^−1^ increases, which may indicate a change of lipids. Similarly, Takahashi et al. [36] studied the changes of OA cartilage by NIR–Raman spectroscopy and found that changes in the amide III bands are different in patients with different stages of OA. The changes in the amide III were derived from structural and direction changes in collagen fiber bundles, and the amide III band ratio (1241/1269 cm^−1^) can be considered to be a sensitive indicator of a disordered knee cartilage collagen secondary structure, which has significance for the early detection of OA. Oshima et al. [131] divided mice into control and model groups and collected Raman spectra from them, examining the amide I, CH_2_ deformation, amide III, phenylalanine, and hydroxyproline peaks. Their results showed that the phosphate and collagen bands in the OA group were significantly different from those in the control group, and a PCA method could successfully distinguish between the two experimental groups.

Fiber-optic Raman technology, Raman probe [132,133,134], Raman arthroscopy, and other related technologies are constantly developing, and their applications in OA are attracting more attention from researchers. Bergholt et al. [135] used fiber-optic Raman technology to quantify the extracellular matrix (ECM) of living-tissue constructs online. In addition, the similarity between natural cartilage and tissue cartilage constructed from living cells was quantitatively evaluated, and the growth ability of living tissues in different cycles was monitored by this technique, which provided theoretical support for the development of engineered cartilage. Esmonde-White et al. [132] carried out Raman signal collection in cartilage, subchondral bone, and cancellous bone and used a human tissue model to determine the influence of cartilage thickness on Raman signal collection, providing support for further study of the Raman probe. Oshima et al. [136] studied the PG content and collagenous fiber arrangement in a cartilage matrix, which may be related to degenerative changes caused by OA. Moreover, they designed an original Raman device for remote sensing in arthroscopic surgery, and a grading system for cartilage defects was defined based on the results of Raman spectroscopy. Furthermore, the Raman detection system for early cartilage degenerative injury was evaluated, which proves that it may be a useful new tool for the diagnosis of OA.

## 5. Summary and Prospect

As a degenerative disease with no specific medical treatment, OA is affecting the health of an increasing number of people worldwide. Early diagnosis can effectively prevent the deterioration in OA, reducing the pain experienced by patients and the cost of treatment. However, there are limitations associated with using complex and expensive imaging methods such as MRI, OCT, and X-ray for early diagnosis. They are commonly used in clinical practice but are often time-consuming, expensive, or even destructive. Vibrational spectroscopy techniques, including NIR, IR, and Raman spectroscopy, have attracted the attention of OA researchers because of their advantages of being fast, nondestructive, and low cost. The characteristics of these three vibrational spectroscopy techniques are summarized in Table 2. Research on the diagnosis of OA by vibrational spectroscopy at the laboratory stage is currently ongoing.

NIR spectroscopy has strong sensitivity and can be used as an effective method for the early diagnosis of OA through the identification of biomarkers. Therefore, qualitative and quantitative analyses of biomarkers are important research topics for the development of NIR spectroscopy in terms of the early diagnosis of OA. Although it is difficult to determine specific cartilage regions under diffuse reflection, NIR spectroscopy can display full spectral signals and can be used for arthroscopic evaluation of articular cartilage status. Further development of the sample collection methodology, in combination with chemometrics and the exploration of more spectral processing methods, will be conducive to its future development [137,138]. Meanwhile, IR spectroscopy based on the strongest fundamental frequency vibration in molecules, especially FTIR spectroscopy technology, is becoming increasingly advanced for the early diagnosis of OA [139]. Some major research technologies, such as IR probe spectral acquisition technology and FTIR–MS imaging technology, are important means of IR spectroscopy in the early diagnosis of OA. Compared with Raman imaging and NIR spectroscopy, FTIRI has the advantage of speed and is most suitable for large-area chemical imaging in unstained tissue sections. Moreover, since the thickness of the tissue section and the infrared light penetration distance are known, it is feasible to use FTIRI for quantitative analysis. Raman spectroscopy has the advantage of being used to perform molecular analysis, but there are technical limitations of this method, such as tissue autofluorescence, low signal intensity, or phototoxic effects, that may occur after prolonged exposure to laser light [140,141]. Solutions to these limitations currently being explored include using low-energy NIR lasers to optimize parameters and establishing standardized protocols for mathematical and computational modeling involving spectral data processing and analysis. In addition, NIR–Raman spectroscopy is one of the best spectroscopic techniques with high specificity of biomolecules. NIR excitation light can selectively resonate with valuable signal molecules to enhance the Raman signals and effectively avoid fluorescence background interference [35]. This method has been used to detect the vibration of biomolecules and reveal highly specific biochemical structures and conformations of tissues and cells. The application of vibrational spectroscopy in the early diagnosis of OA has attracted increased research focus. Although there are still some limitations, it is being actively developed to achieve more accurate, rapid, and nondestructive diagnostic tools.

## Figures and Tables

**Table 1:** Comparison table of application analysis of traditional imaging methods in relation to osteoarthritis (OA).

Name	Main Inspection Site	Advantages	Disadvantages	Reference
MRI	Structure of cartilage	Observe structural features related to cartilage integrity, no radiation	Expensive, long scan time	[15,16,17,18]
OCT	Transverse view of articular cartilage	Good reproducibility and no radiation to living tissue	Depth limitations allow only surface cross-sectional analysis	[18,19,20,21]
Ultrasonic diagnosis	Periarticular soft tissue	Visible image information, low cost	Poor penetration of bone, inability to observe deeper structures, poor reproducibility	[22,23,24,25,26]
X-ray	Joint appearance	Low cost, high benefit, combined with arthroscopy to assist doctors in diagnosis	Unable to show early symptoms of OA	[27,28,29]

**Table 2:** Comparison table of application analysis of vibrational spectroscopy.

Vibrational Spectroscopy	Working Principle	Advantages	Disadvantages	Application in OA
NIR spectroscopy	Reflects the overtones and/or combination bands of stretching and bending vibrations of C–H, N–H, and O–H bonds ranging from 12,500 to 4000 cm^−1^	Fast, accurate, nondestructive, labor-saving	Wide band, high spectral overlap, difficult to distinguish characteristic peak	Has a high penetration depth but can only provide a full spectral signal of the cartilage
IR spectroscopy	Studies the structural changes in the range (4000–400 cm^−1^) caused by the transition of vibrational and rotational energy levels of molecules	Fast, accurate, and nondestructive; reflects information of most organic matter	Low sensitivity, complex band, sample limitations	Detects a variety of components in the cartilage and can achieve high-speed imaging
Raman spectroscopy	Reflects the vibrational information between molecules based on the principle of Raman scattering	Weak water signal, fast, simple, reflects biological signal	Raman scattering area can be affected by the optical system, fluorescence interference	Reflects physiological changes of OA at tissue, cell, and molecular levels
